# Percutaneous Coronary Intervention (PCI) Strategies under Hemodynamic Support for Cardiogenic Shock: A Single-Center Experience with Two Patients

**DOI:** 10.1155/2020/6260239

**Published:** 2020-05-28

**Authors:** Talha Ahmed, Diljon Chahal, Ronson J. Madathil, David Kaczorowski, Anuj Gupta

**Affiliations:** ^1^University of Maryland Medical Center Midtown Campus, USA; ^2^University of Maryland Medical Center, School of Medicine, USA

## Abstract

We describe two cases of profound cardiogenic shock complicating acute myocardial infarction (CSAMI) requiring mechanical circulatory support (MCS) with venoarterial extracorporeal membrane oxygenation (VA-ECMO) allowing complex, high-risk, and staged percutaneous interventions with successful decannulation but with unfortunate outcomes.

## 1. Introduction

Mechanical circulatory support (MCS) is utilized for refractory cardiogenic shock (CS) that does not respond to inotropes and vasopressors. However, evidence for use of this treatment strategy to improve mortality outcomes is limited. In light of current guidelines, MCS with some preference based on availability, institutional experience, presence of hypoxia, and right ventricle (RV) dysfunction should be considered in refractory CS [1, 2]. We describe two cases of refractory cardiogenic shock complicating acute myocardial infarction (CSAMI) requiring venoarterial extracorporeal membrane oxygenation (VA-ECMO) support and left ventricle (LV) venting that allowed for complex and staged percutaneous coronary interventions (PCI). Both patients were eventually weaned off the ECMO but died during the hospitalization.

## 2. Case 1

A 52-year-old male with past history of hypertension, diabetes, stroke, and seizure disorder presented to an outside facility with chest pain, dyspnea, and syncope. His electrocardiogram (EKG) showed anterior ST elevation MI (STEMI). Chest X-ray (CXR) revealed pulmonary edema. Emergent coronary angiography “*first stage*” was performed revealing severe triple vessel disease for which he underwent stenting of the left anterior descending (LAD) artery with a drug-eluting stent (DES). An intra-aortic balloon pump (IABP) was placed due to elevated left ventricle end-diastolic pressure (LVEDP). The patient became progressively hypoxic and was intubated with high requirements for fraction of inspired oxygen (FiO_2_) of 100% and positive end-expiratory pressure (PEEP) of 20 cmH_2_O. He was referred to our hospital for consideration for ECMO support.

On presentation to our hospital, he was on hemodynamic support with inotropes (epinephrine at 0.21 mcg/kg/min) and vasopressors (norepinephrine at 0.38 mcg/kg/min) along with IABP. His troponin I peaked at 444 ng/ml with lactate of 11 mmol/l. Echocardiogram revealed severely decreased left ventricle ejection fraction (LVEF) of 20% with wall motion abnormalities involving LAD territory and normal RV size and function. Due to severe hypoxic respiratory failure and refractory cardiogenic shock, he was cannulated for VA-ECMO at bedside followed by an Impella CP (Abiomed) placement using a right femoral artery approach for LV venting and the IABP was removed. The ECMO inflow cannula (19 French/Fr) was at the left femoral artery while the outflow cannula (25 Fr) at the right common femoral vein with blood flow of 3.16 liters per min.

**Figure 1:**
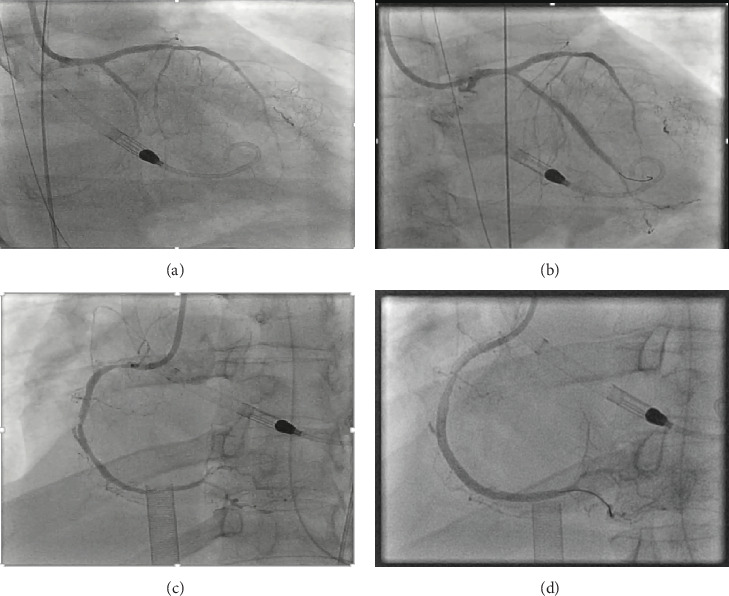
(a) Coronary angiogram showing left coronary artery disease with chronic total occlusion (CTO) of the obtuse marginal 1 (OM1) branch of the left circumflex (LCx) artery. (b) Postcoronary intervention with restoration of TIMI 3 flow in the OM1 branch. (c) Coronary angiogram showing severe diffuse right coronary artery (RCA) disease. (d) Postcoronary intervention with restoration of TIMI 3 flow in RCA.

In the “*second stage*” of PCI, the proximal and distal RCA lesions were predilated and then stented with three DES followed by aggressive postdilation. CTO of mid left circumflex (LCx) extending into the distal obtuse marginal 1 (OM1) was predilated followed by placement of DES. TIMI-3 flow was demonstrated with no evidence of dissection or perforation (Figures 1(a)–1(d)).

The patient's clinical status started to improve and was weaned off inotropic and vasopressor support. He was decannulated from ECMO, and Impella device was removed on the 7^th^ day of admission. He however developed severe pneumonia and had a cardiac arrest for which he was placed back on ECMO with Impella CP (Abiomed) insertion via the left femoral artery approach due to severe hypoxic respiratory failure from pulmonary edema. His lactate trended down to 1.7 mmol/l on day 3 of the first ECMO cannulation and stayed normal after decannulation from ECMO but again trended up to 6.1 mmol/l after the second arrest. He was found to have multiple new strokes on brain imaging, and considering worsening clinical status and poor outcomes, the family decided to withdraw care. The patient died a few hours later as the discussion regarding continuation versus withdrawal of hemodynamic support including ECMO and Impella was being discussed with the family.

## 3. Case 2

A 79-year-old African American male with past history of hypertension, hyperlipidemia, and insulin-dependent diabetes mellitus presented with exertional chest pain and dyspnea that got progressively worse to the point that he presented to an outside hospital. Examination revealed trace pedal edema, and auscultation of chest revealed mild crackles but no murmurs. His EKG and initial troponin I were normal which later peaked at 1.50 ng/ml and trended down. CXR revealed cardiomegaly and mild pulmonary edema. Echocardiogram revealed dilated LV with global hypokinesia (LVEF of 15%) and normal RV size and function. Coronary angiography revealed severe three vessel disease, and he was referred to our facility for coronary artery bypass grafting (CABG) evaluation. On presentation to our hospital, he suffered a pulseless electrical activity (PEA) arrest on day 1 complicated by refractory CS and was cannulated for VA-ECMO. The ECMO inflow cannula (19 Fr) was at the right femoral artery while the outflow cannula (25 Fr) at the left common femoral vein with blood flow of 3.1 liters per min. After initial stabilization, he underwent staged high-risk and complex PCI on days 2 and 10 of presentation.

**Figure 2:**
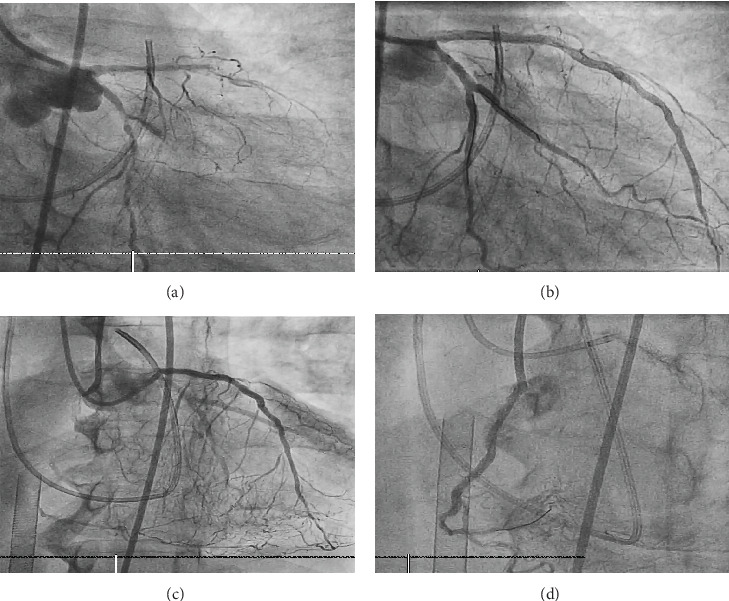
(a) Coronary angiogram showing severe disease in the left anterior descending (LAD) and left circumflex (LCx) arteries. (b) Postfirst stage of coronary intervention with restoration of TIMI 3 flow in the LAD and LCx. (c) Coronary angiogram showing severe diffuse right coronary artery (RCA) disease with CTO. (d) Postsecond stage of coronary intervention with restoration of TIMI 3 flow in RCA.

During the “*first stage*,” 100% occlusion of ostial to distal LAD was managed by successful rotational atherectomy with the deployment of four DES. LCx had severe proximal to mid calcific disease managed by rotational atherectomy and one DES. Large OM2 had CTO treated with an antegrade wire escalation technique and placement of a DES using modified T and small protrusion strategy with final bifurcation kissing balloon inflation (Figures 2(a) and 2(b)). CTO of RCA was seen with retrograde filling via LAD septals to the right posterior descending artery septals. For this reason and also to limit the complications from contrast use, RCA intervention was not performed in the first stage. Given significant pulmonary edema with an elevated LVEDP, an Impella CP (Abiomed) device was placed via the left femoral artery at the completion of the case for LV unloading. Post PCI, stress testing showed viable RCA territory and matched metabolic-perfusion defect in LAD and LCx territory. In the “*second stage*,” CTO at the ostial RCA was managed by successful deployment of two overlapping DES via an antegrade approach guided by intravascular ultrasound (IVUS) (Figures 2(c) and 2(d)). Impella was replaced with left axillary Impella 5.0 (Abiomed) due to anticipated long-term need, with a goal to decannulate from VA-ECMO.

The patient was decannulated from VA-ECMO to axillary Impella on day 14 due to improvement in hemodynamic status. His echocardiogram on day 1 (at time of PEA arrest) showed dilated LV with diffuse hypokinesia (EF of 10%) and nondilated RV with moderately decreased function. A follow-up echocardiogram almost 1 month later showed slightly improved LVEF of 25% with global hypokinesia and normal right RV size and function. The patient however continued to require axillary Impella 5.0 support as well as vasopressor (norepinephrine at 0.14 mcg/kg/min, vasopressin at 0.03 units/min) and inotropic support (epinephrine at 0.05 mcg/kg/min). He developed severe pneumonia and suffered a cardiac arrest after 54 days of admission from which he could not be resuscitated.

## 4. Discussion

Cardiogenic shock (CS) is a state of low tissue perfusion, inadequate to meet the needs of the body that results from primary cardiac dysfunction [1]. The clinical presentation of CS is varied from hemodynamic abnormalities of preshock to mild shock, progressing to more profound shock and finally refractory shock, which is associated with high mortality rates. Acute coronary syndrome continues to be the most common cause of CS, and mortality from CSAMI remains high despite early revascularization and advents in medical therapy [2].

First-line treatment for cardiogenic shock involves hemodynamic support with inotropes and vasopressors [3]. Percutaneous MCS for refractory CS have been in practice for some years now. These include but are not limited to IABP, Impella device, tandom heart, and VA-ECMO [3]. VA-ECMO is most commonly considered for patients with biventricular failure and profound hypoxemia refractory to other medical or device-based intervention [4].

Risk factors associated with worse long-term outcomes after VA-ECMO include increasing age; comorbidities like ischemic heart disease, diabetes mellitus, renal failure, and chronic obstructive lung disease; degree of acid base disturbance; liver/kidney dysfunction; and inadequate unloading at the time of ECMO initiation. Risk scores including ENCOURAGE score, SAVE (survival after VA-EMCO), and the simple cardiac ECMO scores can assess likelihood of survival to hospital discharge with modest discrimination [5–7]. In our cases, patient 1 had a ENCOURAGE score of 28 (with 30-day and 6-month survival post ECMO initiation of 17% and 7%, respectively) and SAVE score of -12/Class V (18% in-hospital mortality) while patient 2 had ENCOURAGE score of 19 (30-day and 6-month survival post ECMO initiation were 35% and 25%, respectively) and SAVE score of -18/Class V (18% in-hospital mortality).

In patients with signs of LV distension and worsening pulmonary edema, some form of LV unloading or venting strategy needs to be introduced. A number of LV venting strategies have been used historically each with its own limitations and advantage. The use of Impella device as LV venting has demonstrated reduction in LV end-diastolic dimension and pulmonary edema while axillary Impella allows for patients' mobility while being on support [8].

According to the 2014 American Heart Association/American College of Cardiology (AHA/ACC) NSTEMI guidelines, a revascularization strategy with multivessel PCI in contrast to culprit lesion-only PCI may be reasonable in patients undergoing coronary revascularization as part of treatment for NSTEMI [9]. This however does not account for patients in cardiogenic shock since results from the CULPRIT-SHOCK trial reveal that in patients with cardiogenic shock complicating myocardial infarction, culprit lesion-only PCI improves outcomes including 30-day mortality and time to renal revascularization therapy as compared to the multivessel approach [10]. The 2018 European guidelines recommend using the revascularization strategy based on clinical status, comorbidities, and disease severity of patients. Similarly in patients with STEMI, current data favors complete revascularization before hospital discharge; however, optimal timing (single stage vs. multiple staged) of procedures should be determined by clinical status of the patient [11].

In a single-center study, Shaukat et al. described high-risk PCI using VA-ECMO support in five patients who presented with either LV systolic dysfunction or NSTEMI [12]. In an interesting observational study involving almost 287 patients from a large registry, Basir et al. concluded that early initiation of MCS even before initiation of inotropes or vasopressors was associated with increased survival in patients presenting with CSAMI. In their study, Impella device was used as MCS [13]. Whether or not these results will hold true for VA-ECMO support has not been evaluated thus far.

## 5. Conclusion

These cases highlighted that MCS can support staged PCI, but we need more data before this can become routine practice. However, great emphasis should be placed on patient selection in order to gain maximal benefit out of these invasive devices while limiting their inherent side effects. This particularly is true for elderly frail patients and those with multiple comorbidities leading to poor prognosis as determined by various risk predictors.

## Abbreviations

MCS: Mechanical circulatory support
CS: Cardiogenic shock
VA-ECMO: Venoarterial extracorporeal membrane oxygenation
RV: Right ventricle
LV: Left ventricle
IABP: Intra-aortic balloon pump
CSAMI: Cardiogenic shock complicating acute myocardial infarction
PCI: Percutaneous coronary intervention
STEMI: ST elevation MI
CXR: Chest X-ray
LAD: Left anterior descending
DES: Drug-eluting stent
LVEDP: Left ventricle end-diastolic pressure
LVEF: Left ventricle ejection fraction
CTO: Chronic total occlusion
LCx: Left circumflex
OM: Obtuse marginal
LHC: Left heart catheterization
CABG: Coronary artery bypass grafting
PEA: Pulseless electrical activity
TAP: T and small protrusion
rPDA: Right posterior descending artery
IVUS: Intravascular ultrasound
SAVE: Survival after VA-ECMO
AHA/ACC: American Heart Association/American College of Cardiology.

## Additional Points

*Learning Points*. (1) The use of MCS in patients with CS has not shown mortality benefit so far but does prove to be substantial in refractory cardiogenic shock. (2) In patients undergoing urgent or emergent VA-ECMO cannulation, various risk predictors can determine in-hospital mortality. (3) Early initiation of VA-ECMO either simultaneously or even prior to inotropes or vasopressors has not been evaluated so far. (4) However, needed evidence and studies are underway to determine the effects of the MCS on both short-term and long-term outcomes as well as their cost-effectiveness in patients with CS (ANCHOR trial and EURO SHOCK trial) [14, 15].
